# *Exophiala dermatitidis* Eye Infection: Case Report and Literature Review

**DOI:** 10.3390/jof12050368

**Published:** 2026-05-16

**Authors:** Suzana Otašević, Marija Trenkić, Marko Stalević, Marina Ranđelović, Slavica Stojnev, Milica Đorđević, Jana Pešić Stanković, Goran Koraćević, Roberta Iatta

**Affiliations:** 1 Department of Microbiology and Immunology, Faculty of Medicine, University of Niš, 18000 Niš, Serbia; marina87nis@gmail.com (M.R.); milica.djordjevic.mikrobiolog@medfak.ni.ac.rs (M.Đ.); janapesic90@gmail.com (J.P.S.); 2 Public Health Institute Niš, 18000 Niš, Serbia; 3 Department of Ophthalmology, Faculty of Medicine, University of Niš, 18000 Niš, Serbia; marija.trenkic@gmail.com; 4 Ophthalmology Clinic, University Clinical Center Niš, 18000 Niš, Serbia; 5 Department of Physiology, Faculty of Medicine, University of Pristina in Kosovska Mitrovica, 38000 Kosovska Mitrovica, Serbia; marko.stalevic@med.pr.ac.rs; 6 Department of Pathology, Faculty of Medicine, University of Niš, 18000 Niš, Serbia; slavicastojnev@gmail.com; 7 Center for Pathology, University Clinical Center Niš, 18000 Niš, Serbia; 8 Department of Internal Medicine and Patient Care, Faculty of Medicine, University of Niš, 18000 Niš, Serbia; gkoracevic@yahoo.com; 9 Interdisciplinary Department of Medicine, University of Bari, 70124 Bari, Italy; roberta.iatta@uniba.it

**Keywords:** exogenous ocular infection, fungal endophthalmitis, *Exophiala* spp., *Exophiala dermatitidis*

## Abstract

*Exophiala* endophthalmitis of exogenous origin is an exceptionally rare but severe ocular infection, characterized by diagnostic delays, limited therapeutic guidance, and frequently poor outcomes. Herein, we report one new case of an 80-year-old woman who presented with severe fungal keratitis progressing to endophthalmitis two years after an uncomplicated cataract surgery. The condition was initially misdiagnosed and treated with topical antibiotics and corticosteroids. By cultivation, microscopy, histopathological, and PCR analysis of the samples, *Exophiala dermatitidis* was identified as the causative agent. Despite targeted antifungal therapy with voriconazole, the disease rapidly progressed, resulting in corneal perforation and evisceration of the affected eye. The number of confirmed cases of this infection remains very limited. To address this gap, we conducted a structured review of all reported instances of exogenous *Exophiala* endophthalmitis, in which *Exophiala dermatitidis* emerged as the predominant causative species. Common predisposing factors included corneal barrier disruption, ocular surgery, diabetes mellitus, and corticosteroid use. Diagnostic confirmation was frequently delayed, and treatment outcomes varied. Amphotericin B-based regimens were associated with poor results, whereas voriconazole, particularly when combined with surgical intervention, demonstrated more favorable outcomes. Exogenous *Exophiala* endophthalmitis remains underrecognized, with limited evidence to guide management. This entity should be considered in postoperative or trauma-associated intraocular inflammation, and current evidence supports azole-based therapy combined with surgical intervention when indicated.

## 1. Introduction

Endophthalmitis is an inflammation of the intraocular fluids (i.e., vitreous and aqueous humor), most commonly caused by infections of bacterial, viral, and fungal origin [1]. Fungal endophthalmitis is a rare but serious condition that can cause significant vision impairment and diminish quality of life, typically occurring as an endogenous infection due to fungemia, frequently caused by *Candida* spp. [2].

Although endogenous endophthalmitis caused by filamentous fungi such as *Aspergillus*, *Fusarium*, and *Scedosporium* species has been reported, primarily in immunocompromised individuals [1], non-dermatophytic molds originating from the environment are associated with exogenous eye infections. Among these, hyalohyphomycetes, particularly *Aspergillus fumigatus* and *Fusarium solani*, are the predominant causes of endophthalmitis [3]. Rarely, ocular infections by *Paecilomyces* spp., *Penicillium* spp., and *Acremonium* spp. are reported in limited geographic areas [4,5]. Additionally, dematiaceous molds, including species of the genera *Exophiala*, *Acremonium*, *Alternaria*, *Bipolaris*, and *Curvularia*, have also been identified as causative agents of exogenous endophthalmitis [2].

The onset of clinical manifestations of fungal endophthalmitis typically includes eye redness and vision loss, often preceded by ocular infections of other origin, surgical procedures, or trauma [3,6]. The primary risk factors include disruption of the corneal barrier, with penetrating trauma and intraocular procedures, such as laser-assisted in situ keratomileusis (LASIK) and penetrating keratoplasty. Exogenous keratitis, other ocular infections [7], soft contact lens use, diabetes mellitus, and advanced age represent additional predisposing factors [4]. In rare instances, outbreaks of post-injection endophthalmitis have been associated with the use of contaminated solutions via intravitreal injections [1].

Fungal endophthalmitis typically follows a subacute course, with symptoms developing over a week to one month [7]. Despite the potential severe complications, both the diagnosis and treatment remain challenging. Diagnosis is guided by clinical history (gradual symptom onset, mild to moderate pain, and progressive visual impairment), as well as the presence of risk factors such as recent ocular surgery, ocular trauma, or immunodeficiency, and is supported by ophthalmological evaluation, including slit-lamp biomicroscopy and B-scan ultrasonography. However, delays in etiological agent identification are common in routine clinical practice, leading to adverse disease progression and poor outcomes [2,8]. While conventional diagnostic methods, such as fungal culture from vitreous or aqueous samples, are characterized by low sensitivity, molecular biology techniques offer improved diagnostic accuracy, albeit available in specialized laboratories [9].

Treatment guidelines for fungal endophthalmitis remain undefined, although antifungal agents such as amphotericin B, azole derivatives like voriconazole, and, more recently, isavuconazole have shown efficacy [3]. Currently, protocols for managing either the endogenous or exogenous clinical forms remain non-standardised. Major uncertainties persist regarding both surgical and medical approaches, including the choice of antifungal agents, appropriate dosages, routes of administration, and the integration of localized treatments with systemic antifungal therapy [2,8].

## 2. Materials and Methods

Given the rarity of exogenous *Exophiala* endophthalmitis and the limited available clinical guidance, a structured literature review was performed to identify published cases of exogenous ocular infection caused by *Exophiala* species with documented intraocular involvement. Eligible reports included cases with involvement of the anterior chamber, aqueous humor, iris, lens, or intraocular lens surface, vitreous, or cases explicitly diagnosed as endophthalmitis by the original authors.

Searches were conducted in PubMed/MEDLINE, Scopus, and Google Scholar. Each database was searched using Boolean operators combining terms “*Exophiala*”, “keratomycosis”, “endophthalmitis”, “fungal endophthalmitis”, “intraocular infection”, and “dematiaceous fungi”, with syntax adopted to each platform. Searches included all articles available up to May 2025. Only publications reporting single cases or case series were considered. No language restrictions were applied. Reference lists of included articles were manually screened for additional relevant studies.

Identified articles were screened sequentially at the title, abstract, and full text levels, and relevant clinical, diagnostic, and therapeutic data were extracted for qualitative synthesis. To complement the literature review, we also report a recent European case of exogenous *Exophiala* endophthalmitis that illustrates the diagnostic and therapeutic challenges identified in the reviewed cases.

## 3. Case History

An 80-year-old female patient was admitted to the Ophthalmology Clinic, University Clinical Center of Niš, Serbia, due to severe pain and loss of vision in her right eye.

In the preceding two months, the patient was treated with topical antibiotics and corticosteroids under the initial diagnosis of keratitis, without clinical improvement. Her medical history included uncomplicated phacoemulsification with intraocular lens (IOL) implantation in the affected eye two years ago, arterial hypertension, and type 2 diabetes mellitus, both managed with oral therapy.

**Figure 1:**
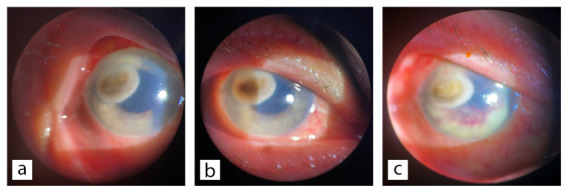
Slit-lamp photographs of the affected eye: (**a**,**b**) Central and paracentral corneal infiltrate with surrounding inflammation; nasal displacement of whitish exudate behind a partially clear cornea. (**c**) Corneal perforation, *Exophiala* spp. stromal infiltrate and hyphema.

At the time of hospitalization, examination of the right (affected) eye revealed light perception without accurate projection and elevated intraocular pressure, estimated by digital palpation. Biomicroscopic evaluation showed ciliary injection, mild conjunctival chemosis, and a full-thickness corneal infiltrate located in the upper temporal quadrant. The infiltrate appeared brownish with irregular borders, penetrating the endothelium, and pronounced folds of Descemet’s membrane were observed. Through the transparent areas of the cornea, whitish, cloudy material was visible on the iris in the nasal half. A hypopyon was present, accompanied by a mild anterior chamber reaction. The pupil was reactively dilated, and the intraocular lens implant was in place. Deeper intraocular structures were not visible (Figure 1a,b). In the left (unaffected) eye, the best-corrected distance visual acuity (BCVA) was 0.6. Senile cataract was noted, and posterior segment examination revealed arteriosclerotic changes, with no evidence of diabetic retinopathy.

Laboratory findings revealed mildly elevated eosinophils (7.46%, reference range 0–6%), elevated erythrocyte sedimentation rate (47 mm/h, reference range 0–15 mm/h), altered glucose metabolism parameters (glucose: 5.5–8.2 mmol/L, reference range 3.9–6.1 mmol/L; HbA1c: 6.7%, reference range 4.2–6.2%), as well as mildly elevated urea (10.2 mmol/L) and creatinine (119.6 µmol/L), while serum iron was slightly decreased (8.3 µmol/L).

Additionally, conjunctival swabs, corneal scrapings from the infiltrate, and aqueous humor were collected and submitted for microbiological analyses.

Empiric treatment was initiated immediately with an intensified antimicrobial regimen. The patient received hourly topical antibiotics (moxifloxacin and tobramycin), nystatin eye drops, anti-glaucoma therapy, and systemic administration of antibiotics (ceftriaxone and gentamicin), along with the antifungal agent fluconazole (IV 200 mg per day). Simultaneously, the anterior chamber was irrigated with antibiotics, and intravitreal injections of ceftazidime (2.0 mg/0.1 mL) and vancomycin (1.0 mg/0.1 mL) were administered.

Although an improvement in visual acuity was observed in the right eye over the next two days, the condition subsequently deteriorated, with progressive corneal edema and the appearance of two whitish masses in the superior part of the anterior chamber. A second anterior chamber washout with antibiotics was performed. B-scan ultrasonography of the posterior segment revealed no signs of vitreous involvement or retinal complications.

### 3.1. Microbiological Examination

**Figure 2:**
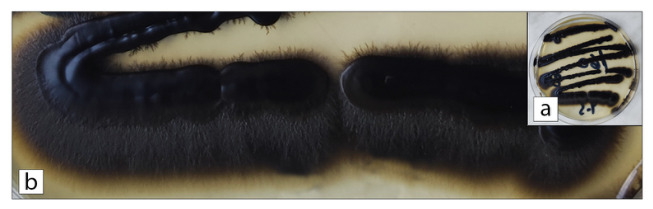
Growth of Exophiala species on Sabouraud dextrose agar: (**a**) Macroscopic appearance of dark brown to black, mucoid, yeast-like colonies with filamentous extensions at the periphery after 7 days of incubation at 28 °C. (**b**) Close-up showing pigmented, velvety colony texture characteristic of dematiaceous fungi (phaeohyphomycetes).

**Figure 3:**
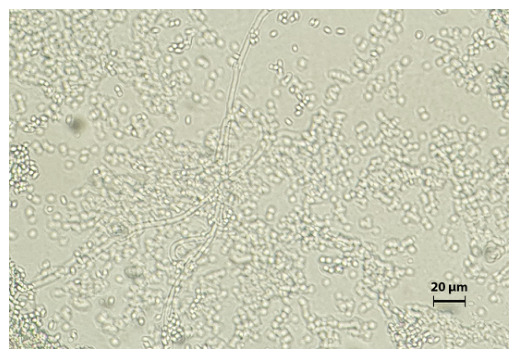
Microscopic examination of isolated fungi showed small yeast-like cells and filamentous elements (magnification ×250).

Initial bacteriological cultures returned negative. However, mycological analysis of the collected samples showed growth of a dematiaceous fungus morphologically identified as *Exophiala* species. Seven days after the cultivation of submitted samples onto both liquid and solid Sabouraud dextrose media (with added chloramphenicol), incubated at 28 °C and 37 °C, the growth of a dematiaceous mold was observed. Macroscopically, the colonies appeared dark brown to black, mucoid in texture, and initially resembled yeast-like growth, with filamentous extensions forming progressively at the colony margins (Figure 2). Microscopically, the fungal structures consisted of subspherical, yeast-like cells and sparsely formed septate hyphae (Figure 3), supporting the identification of Exophiala species.

Antifungal susceptibility testing was performed using the Sensititre™ YeastOne™ YO10 system (Thermo Fisher Diagnostics, Thermo Fisher Diagnostics, B.V., Breda, The Netherlands) according to the recommended protocol for mold testing. It is a commercially available standardized dilution-based antimycogram test with an antifungal panel including the following agents and concentration ranges: amphotericin B (0.12–8 µg/mL), 5-flucytosine (0.06–64 µg/mL), anidulafungin (0.015–16 µg/mL), caspofungin (0.008–8 µg/mL), micafungin (0.008–8 µg/mL), fluconazole (0.12–256 µg/mL), itraconazole (0.015–16 µg/mL), voriconazole (0.06–64 µg/mL), and posaconazole (0.008–8 µg/mL).

The *Exophiala* isolate demonstrated in vitro susceptibility to amphotericin B (MIC = 0.25 µg/mL) and all tested triazoles: fluconazole (MIC = 4 µg/mL), itraconazole (MIC = 0.06 µg/mL), voriconazole (MIC = 0.03 µg/mL), and posaconazole (MIC = 0.03 µg/mL). Resistance was observed to all three echinocandins, with MICs > 8 µg/mL. The MIC for 5-flucytosine was 4 µg/mL, classifying the isolate as susceptible. All MIC values were interpreted according to the Clinical and Laboratory Standards Institute (CLSI) M38-A2 broth microdilution guidelines [10].

### 3.2. The Treatment of Fungal Infection

Following the mycological findings of *Exophiala* species in patient material, systemic antibiotic therapy was promptly de-escalated. In consultation with a clinical pharmacologist and an infectious disease specialist, targeted antifungal treatment was initiated, consisting of intravenous voriconazole (loading dose of 6 mg/kg) and anterior chamber irrigation with voriconazole every 2 days (50 µg/0.1 mL). However, the patient’s clinical condition rapidly deteriorated. Hypopyon and hyphema developed in the anterior chamber, accompanied by a severe inflammatory reaction and the appearance of white deposits on the iris. Visual acuity declined to light perception with inaccurate projection (L+P+/−), followed by corneal perforation (Figure 1c). Given the poor prognosis, evisceration of the right eye was performed.

### 3.3. Histopathological Examination

**Figure 4:**
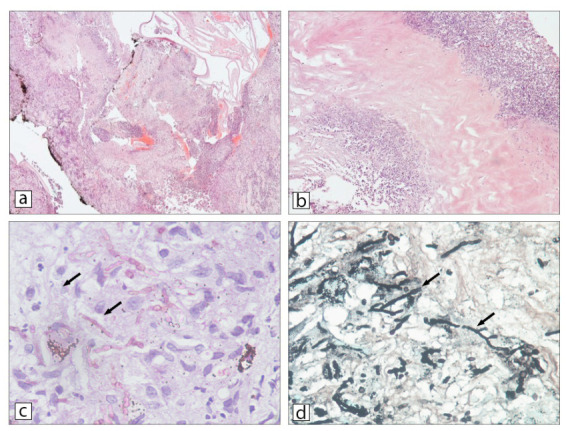
Histopathology of fungal endophthalmitis in eviscerated right eye specimen: (**a**) The internal structures of the eye including uvea with extensive inflammatory infiltration and destructive histological alterations (H&E, ×40). Note the discontinued pigmented layer of the iris; (**b**) Suppurative necrotic inflammation of the cornea (H&E, ×200); (**c**) PAS staining highlights pink fungal hyphae (arrows) (PAS, ×400); (**d**) GMS stains fungal hyphae black (arrows) and budding yeasts. Hyphae are relatively large, septate, and demonstrate progressive dichotomous branching at an acute angle (GMS, ×400). Microscopic examination of isolated fungi showed small yeast-like cells and filamentous elements (magnification ×250).

Histopathological analysis of the eviscerated right eye revealed extensive, destructive inflammation involving the inner layer of the globe. The delicate ocular structures were affected by a dense inflammatory infiltrate composed of histiocytes, multinuclear giant cells of the foreign body type, lymphocytes, neutrophils, and eosinophils. Micromorphological examination revealed poorly organized granulomatous cell aggregates with central necrotizing abscesses, surrounded by marked edema and pronounced vascular congestion (Figure 4a,b). The most prominent pathological changes were observed in the uvea and iris, likely due to their rich vascular supply. Histochemical staining with Periodic acid–Schiff (PAS) and Grocott’s methenamine silver (GMS) enabled visualization of fungal elements within the inflamed tissue. PAS staining revealed pink-staining hyphae (Figure 4c), while GMS staining highlighted black, arborized, twig-like fungal structures with characteristic dichotomous branching at approximately 45 degrees, consistent with filamentous molds (Figure 4d).

### 3.4. Molecular Identification

Genomic DNA was isolated from the fungal sample using the DNeasy Blood and Tissue Kit (QIA-GEN, Hilden, Germany), following the manufacturer’s instructions. The nuclear ribosomal ITS region was amplified using ITS1 (5′-TCCGTAGGTGAACCTGCGG-3′) and ITS4 (5′-TCCTCCGCTTATTGATATGC-3′) primers [11]. The PCR products were purified and sequenced in both directions using the same primers, employing the Big Dye Terminator v.3.1 chemistry in a 3130 Genetic analyzer (Applied Biosystems, Foster, CA, USA) in an automated sequencer (ABI Prism^®^ 377 DNA Sequencers, Applied Biosystems, Foster City, CA,, USA) Nucleotide sequences were edited, aligned, and analysed using Bioedit sequence Alignment Editor 7.0.5.3 [12], and compared with those available in the GenBank using Basic Local Alignment Search Tool (BLAST; National Center for Biotechnology Information (NCBI), National Library of Medicine, Bethesda, MD, USA; http://blast.ncbi.nlm.nih.gov/Blast.cgi; accessed on 20 December 2024). The ITS sequence herein generated showed 100% nucleotide identity with the *Exophiala dermatitidis* CBS125841 strain available in the GenBank database with accession number MH863897.

## 4. Discussion

Exogenous *Exophiala* endophthalmitis is an exceptionally rare clinical entity, with only a limited number of cases described in the literature to date. Among the identified causative species, *Exophiala dermatitidis* is the most frequently documented, with cases reported in North America (USA), Asia, and Europe [8]. Other *Exophiala* species implicated in exogenous endophthalmitis are reported sporadically, reflecting the overall rarity of their ocular involvement.

*Exophiala jeanselmei*, a less prevalent species, has been reported exclusively in the Americas (USA and Brazil) [13,14]. Additionally, isolated case reports of exogenous endophthalmitis due to *Exophiala werneckii* and *Exophiala oligosperma* have been documented in the USA and Africa, respectively [15,16].

The rarity of reported exogenous *Exophiala* endophthalmitis cases observed in our review may partly be a consequence of the fact that some *Exophiala* ocular infections are diagnosed and reported primarily as keratitis, despite evidence of intraocular involvement, potentially leading to misclassification, underrecognition, or lack of reporting.

One such example is the case reported by French authors Benaoudia et al. in 1999 [17], describing an *E. dermatitidis* ocular infection following penetrating keratoplasty. In addition to both cases originating in Europe, this case shares several similarities with the one we present, including postoperative onset, prolonged corticosteroid treatment, predominant involvement of anterior intraocular structures, and poor functional outcome (Supplementary Table S1). While originally classified and presented as keratitis, the authors documented the presence of fungal material on the iris and lens surface, as well as fungal isolation from iris biopsies, all of which suggest spread beyond the cornea into intraocular structures.

Ocular infection caused by *Exophiala* species may manifest as keratitis, subconjunctival mycetoma, or endophthalmitis [8], most commonly as an exogenous infection, following direct inoculation of fungal spores. Reported routes of infection include ocular trauma, ocular surgery such as cataract surgery or keratoplasty, and secondary extension from *Exophiala* keratitis [17], typically affecting immunocompetent individuals.

**Figure 5:**
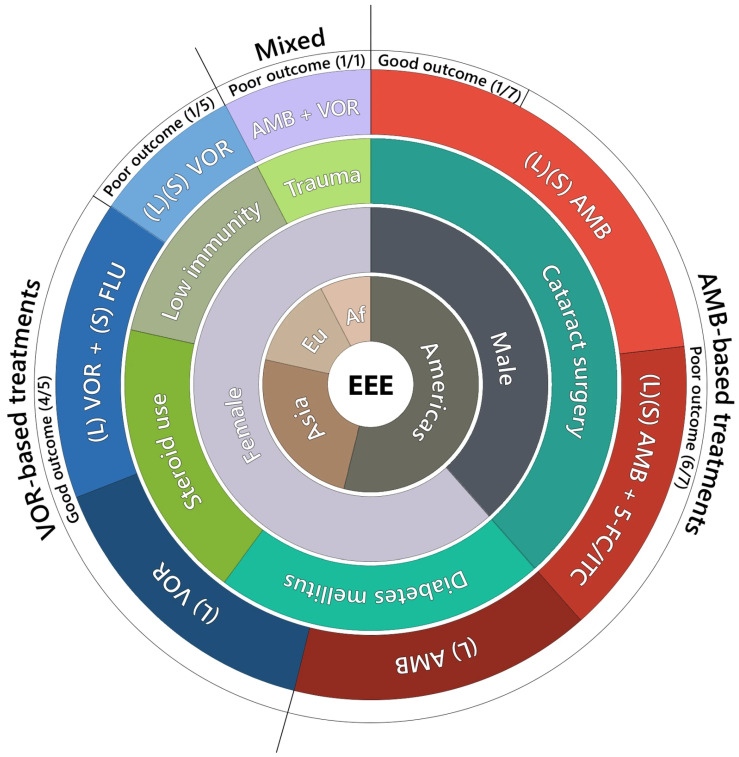
Clinical and Geographic Profile of Exogenous Exophiala Endophthalmitis Cases. EEE—Exogenous *Exophiala* Endophthalmitis (13 cases); Continent: Americas—North and South America (7 cases); Asia (3); Eu—Europe (2); Af—Africa (1); Gender distribution: male (5 cases), female (8); Predisposing factors (one patient may contribute to more than one category): Cataract surgery (8 cases), Diabetes mellitus (5), Steroid use (4), Low immunity—immunocompromised (3), Trauma—local eye trauma (1). Treatment: (L)—Local application; (S)—Systemic application; AMB—Amphotericin B; VOR—Voriconazole; 5-FC—5-Fluorocytosine; ITC—Itraconazole; FLU—Fluconazole: Outcomes: Good outcome: Globe preserved with clinical improvement; Poor outcome: Enucleation; vision loss; eye atrophy.

Although disruption of the corneal barrier is a key initiating event, additional predisposing factors are frequently present, including recent ocular surgery, diabetes mellitus, or long-term corticosteroid therapy (Supplementary Table S1, Figure 5). In the present case, diabetes, prior cataract surgery with intraocular lens implantation, and prolonged use of topical corticosteroids were identified as contributing factors. Other comorbidities documented in the literature include herpes zoster infection and Crohn’s disease [5], both of which may compromise host immune defenses.

Decreased visual acuity, ocular pain, photophobia, and irritation are the most common presenting symptoms of exogenous fungal endophthalmitis caused by *Exophiala* species, and they are generally similar to those seen with other mold-related or intraocular infections. In the present case, initial symptoms included progressive vision loss in the affected eye, accompanied by intermittent pain. Neglected fungal keratitis treated with topical antibiotic and corticosteroid therapy caused a fungal invasion into the interior of the eye.

Ophthalmological examinations revealed ciliary injection, mild conjunctival chemosis, and a full-thickness brownish corneal infiltrate with irregular margins in the superotemporal quadrant, extending into the endothelium. Whitish, cloudy material was visible in the nasal portion of the anterior chamber through a clearer area of the cornea. Additional findings included hypopyon, a mild anterior chamber reaction, and a reactively dilated pupil, with a stable intraocular lens implant. These findings are consistent with previously reported cases of fungal anterior segment involvement, often presenting with severe iridocyclitis or diffuse uveitis accompanied by hypopyon [14,18].

Diagnosis in the present case was established by fungal culture, which enabled the isolation of *Exophiala* spp. from all collected specimens, including a conjunctival swab, corneal scraping, and anterior chamber fluid. However, culture-based identification is inherently time-consuming, and the detection of mold in clinical specimens often raises uncertainty about whether the infection is true versus contamination, thereby contributing to diagnostic delays. Molecular identification directly from clinical material is rarely performed in routine mycological laboratories due to limited availability, cost, and technical requirements. These diagnostic challenges are further compounded by the frequent lack of early clinical suspicion for fungal pathogens, insufficient laboratory infrastructure, the absence of rapid diagnostic tools, and limited access to antifungal agents in routine clinical practice. Although the causative agent was successfully isolated, identified, and subjected to in vitro antifungal susceptibility testing, the prolonged diagnostic process delayed the initiation of targeted local and systemic antifungal therapy.

Similar to the diagnostic challenges, the treatment of endophthalmitis caused by *Exophiala* species remains poorly defined. In the presented case, the isolated *Exophiala dermatitidis* strain demonstrated in vitro susceptibility to amphotericin B and triazoles. Despite in vitro efficacy of voriconazole, combined systemic and local therapy with this antimycotic was ineffective in controlling the infection, and the patient’s condition deteriorated rapidly, ultimately requiring evisceration of the affected eye. One possible explanation for this outcome lies in the general notion that if a fungus is resistant in vitro, a good in vivo effect cannot be expected. However, even when an antifungal agent shows significant in vitro activity, this does not necessarily imply satisfactory in vivo efficacy. Another reason for the lack of voriconazole effectiveness in this patient may be related to the prolonged time required for mold cultivation and identification, as well as susceptibility testing and the delayed initiation of therapy. This was further compounded by the absence of general protocols, guidelines, and clear clinical procedures for initiating antifungal treatment.

An overview of reported cases of exogenous *Exophiala* endophthalmitis, including antifungal treatment strategies and clinical outcomes, is presented in Supplementary Table S1 and summarized in Figure 5. Poor outcomes, such as ocular atrophy, total retinal detachment, or evisceration, were reported in six patients treated with local and/or systemic amphotericin B-based regimens [5,13,14,16,19]. In contrast, cases managed with voriconazole-based therapy, either as monotherapy [18] or in combination with other antifungal agents, such as fluconazole [8,20], were more frequently associated with visual improvement, particularly following surgical intervention such as vitrectomy. Nevertheless, one case was complicated by infection recurrence requiring regrafting after keratoplasty [21].

Regarding antifungal susceptibility, based on our results, *Exophiala dermatitidis* demonstrated good in vitro sensitivity to amphotericin B, voriconazole, and other tested triazoles, consistent with findings from previously published in vitro studies. In contrast, only a few studies have been conducted on susceptibility to echinocandins, with contradictory results, classifying the mold as either sensitive [16] or resistant [8]. In the presented case, the MIC values of all tested echinocandins placed them among ineffective antifungal agents.

## 5. Conclusions

Based on available data from reported cases in the literature, it can be emphasized that in ocular infections with suspected fungal etiology, such as exogenous endophthalmitis and severe keratitis, the diagnostic approach should follow principles established for invasive fungal infections. In this context, the possibility of a fungal etiology should always be considered in high-risk patients, particularly those with diabetes mellitus, a history of cataract surgery or intraocular lens implantation, prolonged use of topical corticosteroids, or trauma. Accordingly, this diagnostic consideration should be communicated to microbiology laboratories and considered during the processing of clinical samples. Although treatment outcomes remain variable, available evidence suggests that voriconazole-based regimens, particularly when combined with surgical intervention, may be more effective than amphotericin B-based regimens.

## Data Availability

All data supporting the findings of this study are included within the article.

## Supplementary Materials

The following supporting information can be downloaded at: https://www.mdpi.com/article/10.3390/jof12050368/s1, Table S1: Reported cases of exogenous Exophiala endophthalmitis.
