# Supplementary material for: Exophiala dermatitidis Eye Infection: Case Report and Literature Review
Source: J Fungi (Basel). 2026 May 16;12(5):368. doi: 10.3390/jof12050368 (PMC13208715; doi:10.3390/jof12050368)
Supplement: Supplementary file 1 [file jof-12-00368-s001.zip › jof-4238895-Supplementary Table S1.pdf]

Supplementary Table S1. Reported cases of exogenous *Exophiala* endophthalmitis.

Authors	Country	Patient Gender (Age)	Predisposing Factors	Symptoms and Signs	Diagnosis Methods	Pathogen	Treatment	Antifungal susceptibility	Outcome
Hammer et al. [13]	USA	Male (31)	Post-traumatic fungal endophthalmitis following penetrating eye injury	NR	Fungal culture (vitreous aspirate)	*Exophiala jeanselmei*	Intravitreal and intravenous AMB, oral 5-FC	NR	Fungal eradication achieved, but vision loss due to total retinal detachment
Margo & Fitzgerald [19]	USA	Female (75)	Cataract surgery, prolonged steroid use	Decreased vision, painful eye	Vitrectomy; fungal culture	*Wangiella* (*Exophiala*) *dermatitidis*	Multiple vitrectomies, intravitreal AMB (5 µg)	NR	Eye enucleated due to pain
Hofling-Lima et al. [14]	Brazil	Female (67)	Corticosteroid treatment	Corneal abscess, severe anterior chamber reaction	Anterior chamber culture	*Exophiala jeanselmei*	Intravitreal AMB (multiple doses), systemic AMB	NR	Ocular atrophy after recurrence
Hofling-Lima et al. [14]	Brazil	Female (52)	Controlled diabetes	Severe iridocyclitis, diffuse uveitis with hypopyon	Anterior chamber and vitreous culture	*Exophiala jeanselmei*	Vitrectomy, systemic and intraocular AMB	NR	Ocular atrophy after recurrence
Benaoudia et al. [17]	France	Male (31)	Penetrating keratoplasty, prolonged steroid use	Ocular pain, corneal infiltrate; anterior chamber involvement	Fungal culture; histopathology	*Wangiella* (*Exophiala*) *dermatitidis*	Systemic and intraocular AMB; surgical intervention (keratoplasty)	Susceptible to AMB, NYS, KET, resistant to 5-FC, MIC not reported	Poor visual outcome (persistent graft edema)
Huber et al. [15]	USA	Female	Cataract surgery	Decreased vision, pain	Vitreous cultures	*Exophiala werneckii*	Vitrectomy, intravitreal AMB, systemic antifungals	NR	Resolution of infection, improvement in visual acuity
Clamp et al. [5]	USA	Male (60)	Crohn's disease, herpes zoster	Persistent intraocular inflammation,	Vitrectomy; vitreous culture, PCR	*Exophiala dermatitidis*	Multiple vitrectomies, intravitreal AMB	NR	Eye enucleated after recurrence

https://doi.org/10.3390/jof12050368

			keratitis, prior keratoplasty	dense vitritis, retinal infiltrates			(2.5µg; 5µg), intravenous AMB		
Quintero-Estades et al. [21]	Puerto Rico	Female (65)	Cataract surgery	Pain, reduced vision, corneal melt	Anterior segment surgery; fungal culture	*Exophiala* spp.	Penetrating keratoplasty, intravitreal VOR (50 µg), topical VOR (1%)	NR	Improved to 20/80, later required regrafting
Homa et al. [8]	India	Male (59)	Post-cataract surgery	Persistent anterior chamber inflammation, fluffy growth on posterior capsule	Vitrectomy; posterior capsule culture, molecular identification	*Exophiala dermatitidis*	Vitrectomy, removal of IOL and capsular bag, intravitreal VOR (100 µg), oral FLU	**MIC values:** AMB (0.33 µg/mL); CLO (0.33 µg/mL), ECO (0.83 µg/mL), FLU (2.67 µg/mL), ITR (0.25 µg/mL), KET (0.25 µg/mL), POS (0.38 µg/mL), VOR (0.25 µg/mL), NAT (8 µg/mL), ECH (>32 µg/mL)	Visual acuity improved to 6/9
Kim et al. [18]	South Korea	Female (70)	Diabetes, cataract surgery	Vision impairment, anterior chamber inflammation, vitreous opacity, hypopyon	Biopsy, fungal culture	*Exophiala* spp.	Vitrectomy, removal of IOL; VOR eye drops (topical, 5%)	NR	Visual acuity improved to 1.0, no recurrence after 6 months
Van der Merwe et al. [16]	South Africa	Female (75)	Diabetes, cataract surgery	Pain, photophobia, corneal infiltrate	Vitreous aspirate, fungal culture, DNA sequencing	*Exophiala oligosperma*	Vitrectomy, intravitreal AMB (5 µg), topical AMB (0.15%), oral VOR (200 mg twice daily)	**MIC values:** VOR (0.5 µg/mL); ITR (0.25 µg/mL); AMB (0.25 µg/mL); CAS	Eye enucleated after deterioration

https://doi.org/10.3390/jof12050368

								(0.25 μg/mL); NAT (0.25 μg/mL).	
Shah et al. [20]	India	Male (64)	Diabetes, complicated cataract surgery	Pain, redness, reduced vision	Biopsy; fungal culture; lactophenol blue staining	*Exophiala dermatitidis*	Vitrectomy, intravitreal VOR (100 μg); oral FLU (150 mg twice daily)	NR	Visual acuity improved to 6/9
Present case	Serbia	Female (80)	Diabetes, cataract surgery with intraocular lens implantation, prolonged steroid use	Pain, redness, progressive vision loss, corneal infiltrate, hypopyon	Conjunctival swab; corneal scraping; anterior chamber fluid	*Exophiala dermatitidis*	Systemic VOR; repeated anterior chamber irrigation with VOR	**MIC values:** AMB (0.25 μg/mL); FLU (4 μg/mL); ITR (0.06 μg/mL); VOR (0.03 μg/mL); POS (0.03 μg/mL); 5-FC (4 μg/mL); ECH (>8 μg/mL).	Eye enucleated after deterioration

Table legend: NR, not reported; IOL, intraocular lens; MIC, minimum inhibitory concentration; AMB, Amphotericin B; NYS, nystatin; CLO, clotrimazole; ECO, econazole; FLU, fluconazole; ITR, itraconazole; KET, ketoconazole; POS, posaconazole; VOR, voriconazole; NAT, natamycin; 5-FC, 5-fluorocytosine; CAS, caspofungin ECH, echinocandins (caspofungin, anidulafungin, micafungin)

https://doi.org/10.3390/jof12050368
